# Miraculous Recovery After Penetrating Trauma to the Mediastinum

**DOI:** 10.7759/cureus.11106

**Published:** 2020-10-23

**Authors:** Allen Mao, Hunaid N Rana, William Janika Brackett, Suzy Figarola, Samuel McQuiston

**Affiliations:** 1 Radiology, University of South Alabama College of Medicine/USA Health University Hospital, Mobile, USA; 2 Radiology, USA Health University Hospital, Mobile, USA

**Keywords:** penetrating trauma, foreign body, mediastinum

## Abstract

Traumatic injury to the mediastinum can damage critical surrounding structures, including the pericardium, aorta, and bronchial tree. We highlight a miraculous case of a 13-year-old female with no past medical history who presented to the emergency department after being impaled in the chest by a metal fence post. After median sternotomy, the foreign object was removed, and the patient fortunately recovered with no permanent sequelae. The radiographic features of the injury are described, and potential unseen cardiovascular and respiratory complications are discussed.

## Introduction

The mediastinum is a predominant part of the thoracic cavity and houses many vital visceral and vascular structures, including the heart, trachea, lungs, esophagus, and great vessels. Traumatic injury to any of the above can lead to immediate life-threatening demise with hemodynamic collapse. While gunshot and stab wounds are the most common causes of penetrating thoracic trauma, other methods to consider are impalement by sharp objects after an accidental fall. In the event of a history of known penetrating injury, a high index of clinical suspicion should be maintained, and trauma workup should be initiated to rule out injury to mediastinal organs. Cardiovascular and respiratory complications of penetrating trauma to the mediastinum may include pneumothorax, cardiac tamponade, and pneumomediastinum.

## Case presentation

A 13-year-old Caucasian female with no significant past medical history presents to the emergency department with significant chest pain after being impaled by a metal post. She states that she was climbing a fence when she slipped and fell directly on top of the post. Emergency medical services detached the post from the fence. On arrival, she was hemodynamically stable with a Glasgow Coma Scale (GCS) of 15. The patient endorsed severe pleuritic chest pain and lightheadedness but had equal breath sounds bilaterally and was saturating well on room air.

**Figure 1:**
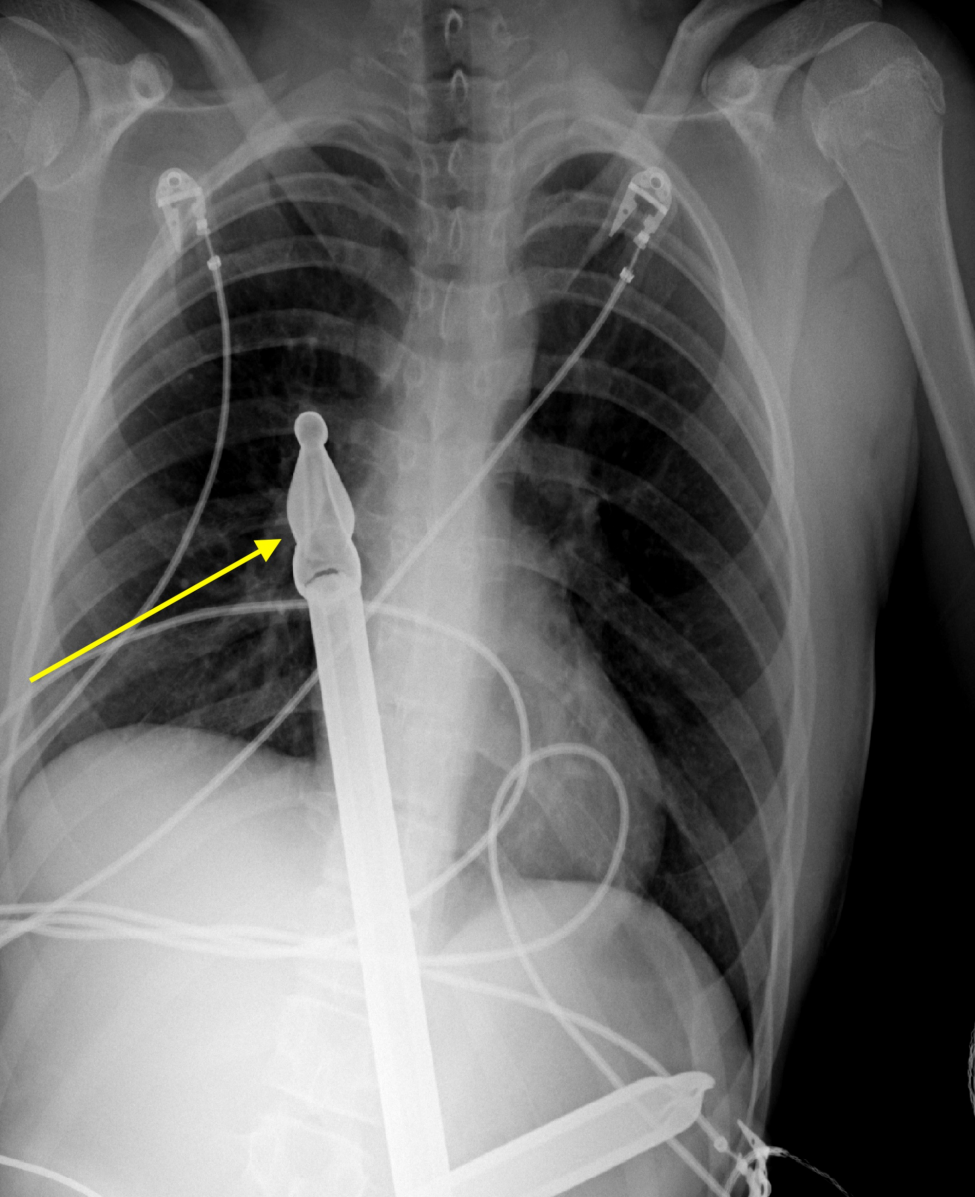
Frontal chest radiograph demonstrates a large foreign body (yellow arrow) consistent with a metal fence post projecting into the mid-right hemothorax

**Figure 2:**
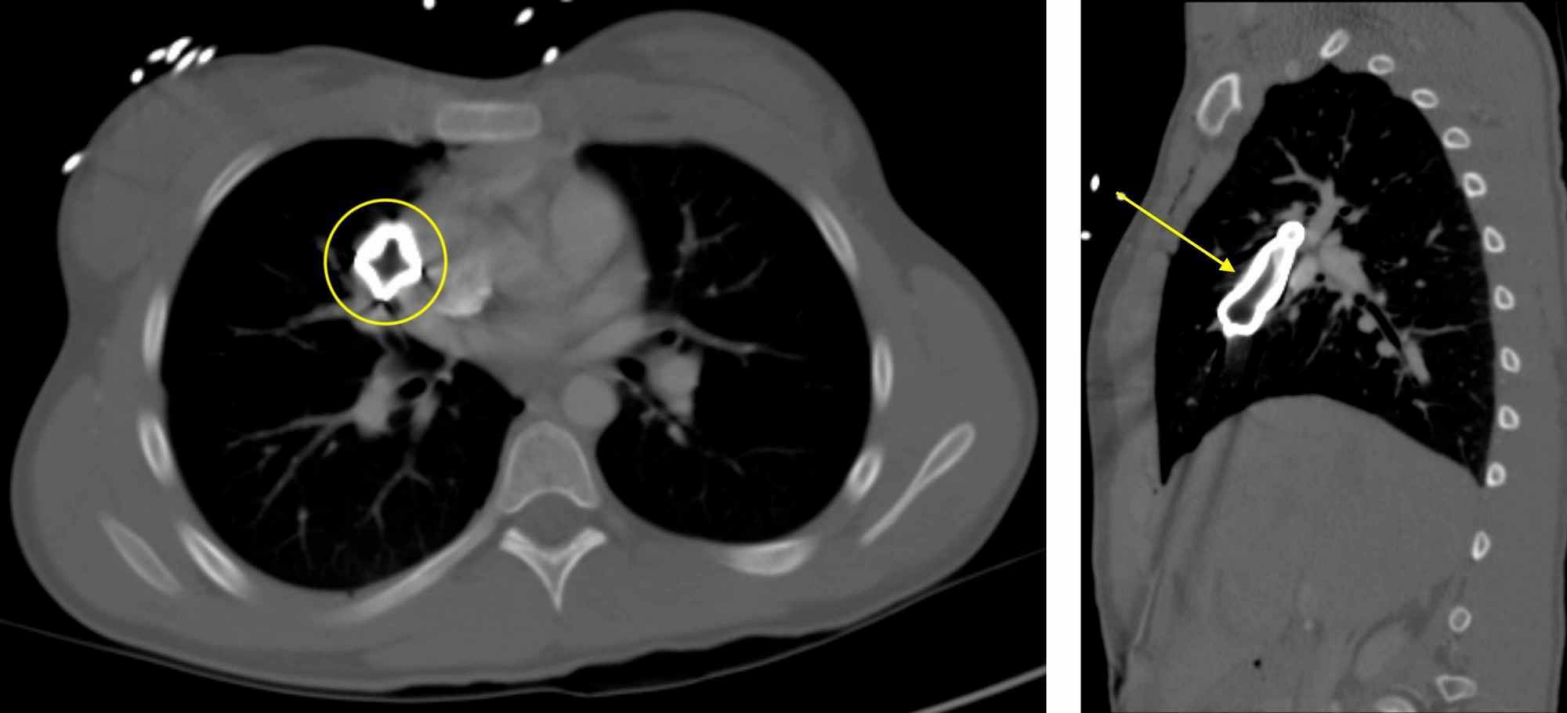
Axial and sagittal CT chest with intravenous contrast display a large retained metallic foreign body (yellow) that penetrates the right chest wall in an anterior to posterior trajectory and extends superiorly through the mediastinum and right hemithorax abutting the adjacent right heart border, hilum, and vasculature

Trauma workup was initiated to determine the extent of internal injuries. The chest X-ray demonstrated a large metallic foreign body projecting into the right hemithorax with no mediastinal widening or pneumothorax (Figure 1). Further characterization with CT chest showed the metallic object entering superolaterally through the anterior-inferior mediastinum and right hemithorax terminating near the right heart border and hilum (Figure 2). The aorta was normal in contour and caliber. The cardiovascular surgery team was consulted for the evaluation and management of the injury.

**Figure 3:**
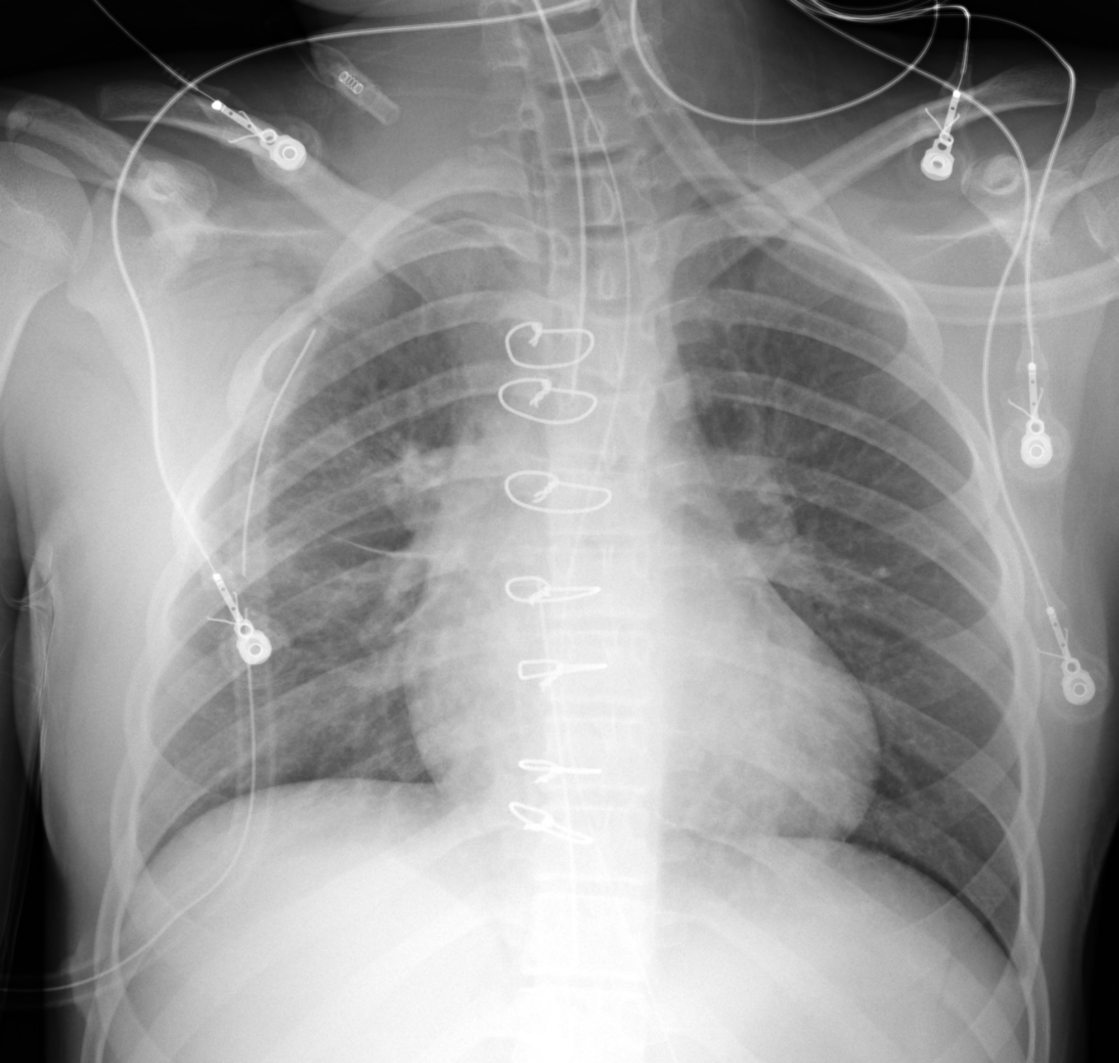
Postoperative frontal radiograph demonstrates interval postsurgical changes of the removal of a large foreign body with midline sternotomy wires, right thoracostomy tube projecting in the right apex, and midline mediastinal drain. The endotracheal tube is seen approximating the carina.

Due to the penetrating injury and concern for damage to vital structures such as the ventricles or right hilum, a median sternotomy was emergently performed in order to remove the foreign body from the thoracic cavity. On surgical exploration, there was penetrating trauma to the mediastinum just superficial to the pericardium and hilum. Miraculously, the foreign body did not pierce the ventricles or the lungs due to a continuous piece of metal crossbar that ran perpendicularly, preventing further entry. The metallic rod measured approximately 45 cm in length. Right thoracostomy and mediastinal tubes were placed in order to drain serosanguinous fluid and postoperative air (Figure 3). The patient was closely monitored in the surgical intensive care unit (ICU). On postoperative day 2, chest tubes were removed, and she was transferred to the floor. Her sternal discomfort was well-controlled with multimodal pain medications. The following day, the patient was asymptomatic and back to her baseline and safely discharged home with close follow-up with cardiovascular surgery as an outpatient.

## Discussion

Cardiovascular and respiratory compromise are concerning complications of penetrating trauma to the mediastinum. Injury to the lungs can lead to a pneumothorax, where air is introduced into the pleural space leading to lung collapse. A chest radiograph can establish the diagnosis and demonstrates a linear shadow of visceral pleura that lacks lung markings peripherally. Management depends on the patient’s hemodynamic stability and size of the pneumothorax. In a clinically stable patient, conservative management is appropriate, with supplemental oxygen and analgesics for pain. On the other hand, hemodynamically unstable patients have decreased venous return to the heart, causing hypotension, and are treated urgently with needle decompression or thoracostomy tube placement [1]. After the procedure, patients are observed closely with serial chest X-rays to monitor for resolution.

Similarly, damage to the heart can result in cardiac tamponade, where blood fills the pericardial sac, causing a pericardial effusion. Patients will typically present with sudden-onset dyspnea, orthopnea, chest pain, and anxiety. Classically, Beck’s triad of hypotension, jugular venous distention, and muffled heart sounds is described. Pulsus paradoxus is another commonly described association with tamponade and presents as an exaggerated decrease in systolic blood pressure > 10 mmHg on inspiration. On electrocardiogram (EKG), patients can present with low voltage in all leads and electrical alternans with varying amplitudes of the QRS complex due to the swinging motion of the heart in pericardial fluid [2]. The effusion can be definitively diagnosed with an echocardiogram that shows the collapse of the right atrium and right ventricle chambers and can measure the quantity of pericardial fluid [3]. In addition, chest radiographs can support the diagnosis by showing an enlarged cardiac silhouette with a possible epicardial fat pad sign, which is consistent with a pericardial effusion [4]. However, plain chest films may not demonstrate an abnormality, especially early in the diagnosis. Rather, chest radiographs are extremely useful in diagnosing additional complications of trauma like pneumothorax or pneumomediastinum. The initial treatment of cardiac tamponade is an emergent pericardiocentesis for the quick removal of fluid, but if the effusion is refractory or recurrent, a subxiphoid pericardial window can be surgically performed to drain the fluid into the pleural cavity.

Pneumomediastinum can be seen following motor vehicle accidents. The most common presenting symptom is chest pain, which is typically pleuritic and retrosternal in nature. There can be associated subcutaneous emphysema in the neck. Hamman’s sign can be seen in approximately 18% of patients and is characterized by a crunching sound heard over the precordium that is synchronous with each heartbeat [5]. On chest X-ray, there will be linear lucencies adjacent to the mediastinum, indicating gas that surrounds mediastinal structures. Patients with isolated pneumomediastinum may be treated conservatively with rest and analgesia and will resolve within a few weeks [6].

## Conclusions

Penetrating trauma to the mediastinum can be life-threatening due to damage to vital organs such as the heart and lungs. We highlight an interesting case of a 13-year-old female who survived accidental impalement by a metal fence rod status-post median sternotomy with the removal of the foreign body. Miraculously, there were no injuries to the ventricles or bronchi, and she underwent complete recovery.
